# Photocatalytic activity and antibacterial properties of linen fabric using reduced graphene oxide/silver nanocomposite

**DOI:** 10.1039/d0ra07544b

**Published:** 2020-11-13

**Authors:** A. Farouk, S. El-Sayed Saeed, S. Sharaf, M. M. Abd El-Hady

**Affiliations:** National Research Centre (Scopus Affiliation ID: 60014618), Textile Research Division 33 El-Behoth Street, Dokki, P.O. Box 12622 Cairo Egypt; Department of Chemistry, Faculty of Science, King Khalid University P.O. Box 9004 Abha Saudi Arabia; Department of Chemistry, College of Science, Qassim University P.O. Box 6666 Buraidah 51452 Saudi Arabia s.saeed@qu.ed.sa; Department of Physics, College of Science and Arts, Qassim University P.O. Box 6666 Al Asyah Buraidah 51452 Saudi Arabia

## Abstract

Silver nanoparticles were *in situ* prepared on the surface of linen fabric coated by graphene oxide (GO). In the meantime, the reduction of silver nitrate on the GO-coated fabric led to the synthesis of reduced graphene oxide on the fabric. Two kinds of substrate (cotton and linen) were used. Both RGO/Ag and Ag/GO nanocomposites were added on cotton and linen fabrics through a conventional “pad–dry–cure” method. The chemistry and morphology of the coated surfaces were extensively characterized using Fourier-transformed infrared spectroscopy, energy-dispersive X-ray spectroscopy, and scanning electron microscopy. Resistivity measurements were used for assessing the conductivity. The UV protection properties and the photocatalytic activity of the coated fabrics against methylene blue dye were also investigated. The antibacterial activity was studied against Gram-positive *S. aureus* and *B. subtilis* and Gram-negative bacterial strains *E. coli* and *P. aeruginosa* by determining the zone of inhibition using the agar diffusion method. Methicillin-resistant *Staphylococcus aureus* (MRSA) has been responsible for many serious hospital infections worldwide. The fabrics showed superior antibacterial activity and successfully hindered the growth of pathogenic bacterial strains. This outcome suggested that both the RGO/Ag and Ag/GO nanocomposites-coated fabrics could be potentially applied in biomaterials and biomedical fields.

## Abstract 2

Silver nanoparticles were *in situ* prepared on the surface of linen fabric coated by graphene oxide (GO).

## Graphical abstract

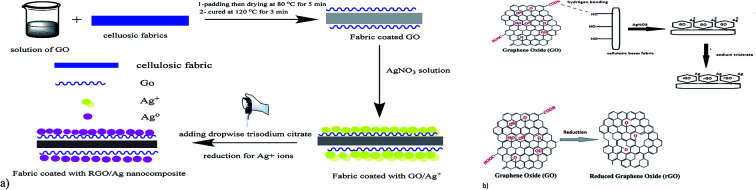

## 1. Introduction

The role of nanotechnology in the fabric industry has arisen due to its outstanding properties. There is an extensive potential for the beneficial utilization of nanotechnology in cotton and other material enterprises. Its application can financially broaden the properties and estimations of textile processing and products. The utilization of nanotechnology enables the multifunctionality of textiles and allows the production of special functional fabrics,^1^ including antibacterial,^2,3^ UV protection,^4,5^ easy-clean,^6^ water- and stain repellent, and anti-odor.^7^

Graphene oxide (GO) is a graphite two-dimensional (2D) form and has lately attracted great interest because of its remarkable properties, including thermal, mechanical, and electronic. The reactivity of GO nanosheets is due to the comportment of a considerable number of hydrophilic functional groups along the surface. Additionally, the oxygen atoms in GO nanosheets are present in a conjugate arrangement that give the GO an electrical insulation character that restricts its utility in electronic areas.^8–11^ Moreover, it can work as new nanoscale building units to form distinctive electroconductive materials due to its high electrical conductivity. It has been used on paper-like materials^12,13^ or dispersed throughout a polymeric matrix^14,15^ to make electrically conductive composites. Therefore, it can be utilized as an electroconductive coating on cellulosic-based textile materials.

Recent research work has focused on developing new-generation nanocomposites through the combination of two distinct functional nanomaterials into one material for diverse applications. In this concept, graphene oxide is an excellent applicant as a supporting material for the metal nanoparticles to form nanocomposites^16^ due to its functional groups containing oxygen, which play a key role in stabilizing the metal nanoparticles and hindering their aggregation. In addition to the presence of hydroxyl, carbonyl, epoxide, and carboxyl groups, the GO sheets form a colloidal stable suspension in pure water, which facilities obtaining different GO-based nanocomposites. GO and silver nanoparticles (AgNPs) with special characteristics have the ability to form nanocomposites with great potential to provide a synergistic and versatile impact that means they can be applied in sensors, catalysis, biomedical, and pharmaceutical fields.^17,18^ Various silver/graphene-based nanocomposites have been prepared to enhance certain properties.^19^ Tang *et al.*^20^ reported the synthesis of high-performance antibacterial agents based on GO/silver nanocomposites with a species-specific mechanism. Moreover, Shahriary *et al.*^21^ investigated the synthesis of a novel glucose sensor through the electrochemical deposition of silver/silver oxide on reduced GO. Recently, Sawangphruk *et al.*^22^ reported coating flexible carbon fiber paper by silver nanoparticle polyaniline–graphene nanocomposites for potential application as a supercapacitor. Ouadil *et al.* reported coating knit polyester fabric with graphene oxide, graphene, and graphene/silver nanocomposites for attaining UV protection, mechanical, and electrical properties.^23^

Electroconducting cellulose-based fibers have been developed as excellent candidates for a wide range of innovative applications in the fields of organic electronics, sensors, anti-statistic materials, electromagnetic inductive inductor harnessing, heating, and energy storage systems.^24^ When compostable or incinerated with energy for recycling at the end of their life cycle, they have the benefits of being plentiful, sustainable, biodegradable, and carbon dioxide free. It takes little energy for their processing and their waste stream is recyclable and biodegradable. Lignocellulose fibers, such as flax and hemp, also have high tensile strength and rigidity, consistent with the existence and crystallinity of cellulose. These fibers are being examined as eco-friendly and cost-effective alternatives to glass fibers and other artificial fibers for strengthening polymers and concrete composites.^25^

Linen fabrics (fax 100%) provide optimum wearing comfort. This is due to their unique characteristic, *i.e.*, cool and wonderful touch, high hydrophilic character, outstanding air permeability, and low agglomeration of electrostatic charges on the textile surface.^26^ Linen has the best thermal conductivity of all natural and chemical textiles; it does not melt. It is also resistant to organic solvents, oxidants, reduced agents, and caustic alkalis, but it is vulnerable to acids and photodegradation.

To the best of our knowledge, no work has yet been done on the treatment of linen fabric with either reduced graphene oxide/silver nanoparticles (RGO/Ag) or silver nanoparticles/graphene oxide (Ag/GO) nanocomposites. Consequently, in this work, we report not only the deposition of Ag/GO nanocomposite on the surface of cellulosic fabrics (cotton and linen) by a traditional “pad–dry–cure” technique but also the *in situ* formation of RGO and Ag nanoparticles on linen as well as cotton fabrics in the same step. Many functions are acquired by both fabrics, but not to the same degree. Additionally, this work clarified the extent of the response of both fabrics to such treatment and its effect on different functions added to the fabrics. The structures of various samples, including a control cotton (C), control linen (L), cotton–GO (C–GO), linen–GO (L–GO), cotton–Ag (C–Ag), linen–Ag (large), cotton–RGO–Ag (C–RGO–Ag), linen–RGO–Ag (L–RGO–Ag), cotton–Ag–GO (C–Ag–GO), and linen–Ag–GO (L–Ag–GO), were characterized by FTIR and SEM, *etc.* Moreover, the photocatalytic properties, UV-radiation protection, and electrical conductivity of the coated fabrics were evaluated, and the mechanism was further investigated.

## 2. Experimental

### 2.1 Materials

Bleached plain woven 100% cotton fabric (138 g m^−2^) and bleached plain weave linen (120 g m^−2^) were supplied by Misr Company for spinning and weaving (Mehalla El-Kobra, Egypt). Graphene oxide, silver nitrate, and trisodium citrate were supplied by Sigma-Aldrich. Methylene blue was supplied by Ciba. All other chemicals and reagents were of analytical grade.

### 2.2 Coating of the fabrics by GO

An aqueous suspension of graphene oxide containing (1 g l^−1^, 50 ml) was prepared under sonication for 2 h. Then, the fabric samples (specimen 10 cm in length and 5 cm in width) were coated by GO solution using a pad–dry–cure method. The fabrics were immersed in pre “suspended” solutions and kept there for 5 min. The samples were then padded in two dips and nips to a wet pick-up of about 100%. After padding, the fabrics were dried at 80 °C for 5 min and cured at 120 °C for 3 min. The GO-coated fabric was taken from the solution and washed with deionized water for the removal of any residual GO on the surface. Finally, all the samples were washed with deionized water several times and then dried. The cotton fabric was coded as C–GO and the linen was coded as L–GO.

### 2.3 *In situ* preparation of silver nanoparticles on the textile fabric

Fabric samples were immersed in a solution of silver nitrate (5 g l^−1^, 50 ml). After that, trisodium citrate (50 g l^−1^, 10 ml) was added dropwise into the reaction mixture with stirring for 30 min at 90 °C.^27^

### 2.4 *In situ* preparation of fabrics coated by GO with silver nanoparticles

GO-coated samples were immersed in a solution of silver nitrate (5 g l^−1^, 50 ml). After that, trisodium citrate (50 g l^−1^, 10 ml) was added dropwise into the reaction mixture with stirring for 30 min at 90 °C. Then, the fabrics coated by RGO/silver nanocomposites were washed several times with deionized water and then dried at 80 °C for 2 h.

### 2.5 Coating of silver nanoparticles-treated fabric by GO

AgNPs-treated samples were immersed in pre-suspended solutions of GO (1 g l^−1^). The samples were then padded in two dips and nips to a wet pick-up of 100%. After padding, the fabrics were dried at 80 °C for 5 min and cured at 120 °C for 3 min. Now the samples had been coated by Ag/GO nanocomposites.

## 3. Characterization

### 3.1 Fourier-transformed infrared spectroscopy (FT-IR)

FTIR spectroscopy has been extensively used in cellulose research since it presents a relatively easy method for obtaining direct information on chemical changes that occur during various chemical treatments. An ATR-FTIR instrument (JASCO, Model IR 4700 Japan) was used, scanning from 4000 to 400 cm^−1^ in ATR mode using KBr as the supporting material. The software was set up to scan the background and samples at a certain number of scans (64), and certain resolution (4).

### 3.2 Scanning electron micrograph SEM/EDX analysis and visual color analysis

SEM/EDX was performed using an FEI INSPECT scanning electron microscope system (Philips, Holland) for environmental scanning without a coating. Elemental micro-probe and elemental distribution mapping techniques were used for analyzing the elemental constitution of the solid samples. The SEM system equipped with an energy-dispersive spectroscope (EDX) was used to perform a rapid quantitative and qualitative analysis of the elemental composition through elemental analysis of the particles.

### 3.3 Antibacterial test

The antibacterial activity of the treated samples against *Staphylococcus aureus*, *Bacillus subtilis* (G +ve), and *Escherichia coli*, *Pseudomonas aeruginosa* (G −ve) bacteria were determined using an agar plate. The antibacterial activity of the fabric samples was evaluated using (ATCC 1533) bacteria with the disk diffusion method. A mixture of nutrient broth and nutrient agar in 1 l distilled water at pH 7.2 as well as the empty Petri plates were autoclaved. The agar medium was then cast into the Petri plates and cooled in a laminar airflow. Approximately 105 colony-forming units of bacteria were inoculated on plates, and then 292 cm^2^ of each fabric sample was planted onto the agar plates. All the plates were incubated at 37 °C for 24 h and were examined to see if a zone of inhibition was produced around the samples.

### 3.4 UV properties

Ultraviolet protection factor (UPF) was measured using an ultraviolet JASCO model V-750 UV/Vis spectrophotometer. UV protection and classification were evaluated according to AS/NZS 4399:1996 with a scan range of 200–600 nm.

### 3.5 Thermal analysis (TGA)

Thermogravimetric analysis (TGA) was carried out using a PerkinElmer TGDTA analyzer, model Pyris 1, operating under a nitrogen atmosphere with initial sample weights of 8 mg. The runs were performed over a temperature range of 50–600 °C at a heating rate of 10 °C min^−1^ under a continuous N_2_ flow of 100 ml min^−1^.

### 3.6 Electrical conductivity properties

The electrical conductivity of the dried fabrics composite was determined at ambient room temperature (25 °C) using a digital multimeter. Electrical measurements were recorded by means of an electrical circuit composed of a Hewlett Packard 6634B system DC power supply and a digital Hewlett Packard 34401A multimeter. The conductivity is given by,Conductivity = 1/*R*_s_where *R*_s_ is the surface resistance. The surface resistance was measured according to the American Association of Textile Chemists and Colorists Test Method 76-1995. All the resistances were determined by averaging 6 measured values on each sample surface. A lower resistance means higher conductivity. Two rectangular copper electrodes (20 × 30 mm^2^) separated by 20 mm were placed on the fabric sample (30 × 60 mm^2^) by a 1 kg mass. The surface resistance (*R*_s_) is given by:
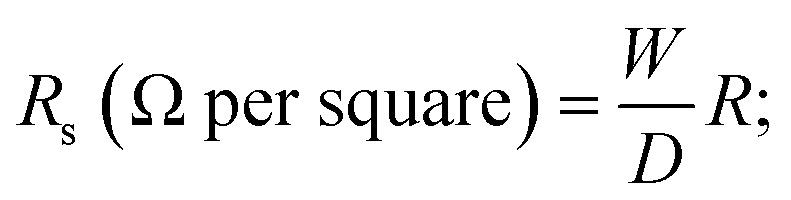
where *R* is the resistance measured in ohm, and *W* and *D* are the width of the sample and the distance between the two electrodes, respectively.

### 3.7 Photocatalytic studies

Evaluation of the photocatalytic degradation of methylene blue (MB). The degradation of adsorbed MB on the coated fabrics was investigated. In addition, the degradation of MB on the coated fabrics coated under different conditions was investigated. In detail, pieces of treated cotton fabric (0.5 g) were placed in separate 100 ml beakers containing 50 ml of aqueous solutions of MB (10 mg l^−1^). The beakers were then exposed to normal laboratory environmental conditions for 24 h under shaking. The rate of decolorization of the colorant solutions was recorded according to the change in the intensity of the absorption peak of MB in the visible region.^28^ UV-vis absorption spectra of the colorant solutions with the treated cotton fabric were recorded using a PerkinElmer Lambda 3B UV-vis spectrometer. For comparison, the same test was also performed using untreated cotton fabric.

### 3.8 Tensile strength

The ASTM test method D-1682-94 (1994) was used to determine the tensile strength of the fabric samples. Two specimens for each treated fabric were tested in the warp direction and the average value was recorded to represent the fabric-breaking load (Lb).

### 3.9 Statistical analysis

The results are expressed herein as mean values with the standard deviation (mean ± S.D.) of each sample from tests repeated three times (*n* = 3). Statistical analysis was performed with the Student's *t*-test and differences were considered as significant at *p*-values below 0.05.

## 4. Results and discussion

### 4.1 Mechanism of coating the fabric by the RGO/Ag nanocomposites

**Fig. 1:**
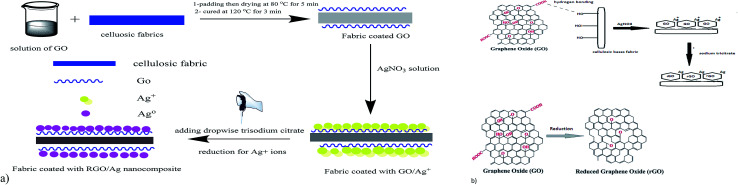
Schematic mechanism for the coating process of cellulosic fabrics: (a) illustration of the coating process and (b) chemical mechanism for the coating process.

The mechanism of coating the fabric with GO is suggested in Fig. 1a and b as follows: the hydrophilicity of GO is due to different functional groups, such as carboxyl, carbonyl, hydroxyl, and epoxy groups, which make the GO soluble in water at molecular levels with a high surface adsorption capability, this allows a strong adherence of GO onto the surfaces of the fabrics.^29^ When the fabrics are immersed in the aqueous solution of GO, a thin coating film of GO immediately strongly adheres to the fabric surface. This could be attributed to the formation of hydrogen bonds between carboxyl and hydroxyl groups of GO and hydroxyl groups of the cellulosic fabrics. GO sheets have a high specific surface area, which makes them an outstanding platform for stabilizing silver nanoparticles.^30^ Upon immersing the fabrics in a solution of silver nitrate, electrostatic interaction facilitates the attraction between the negatively charged oxygen atoms of hydroxyl (OH^−^) and carboxyl (COO^−^) groups of the GO-coated cellulosic fabrics and the positively charged silver ions (Ag^+^). Under heating and stirring conditions, trisodium citrate permits the formation of silver nanoparticles by the concurrent *in situ* reduction of Ag^+^ ions to Ag^0^. In the meantime, GO also will be *in situ* reduced to reduced graphene oxide (RGO). Additionally, the silver nanoparticles are homogeneously distributed between the RGO sheets coated on the fabrics, and the probability of their agglomeration significantly is thus reduced.^31^

### 4.2 FTIR analysis

**Fig. 2:**
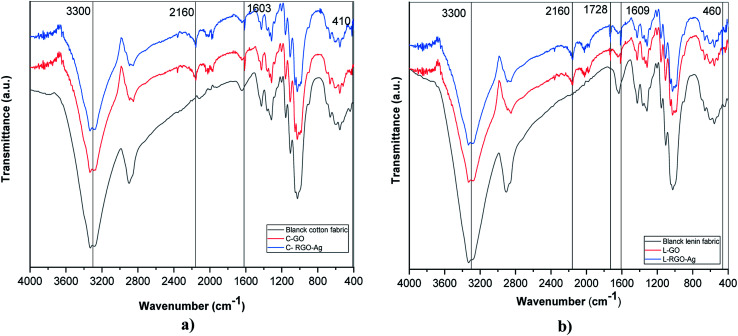
FTIR spectra of the treated fabrics. (a) Spectra for: blank cotton (B-cotton), GO–cotton (C–GO), and RGO–Ag cotton (C–RGO–Ag). (b) Spectra for: blank linen (B-linen), GO–linen (L–GO) and RGO–Ag linen (L–RGO–Ag).

Fourier-transform infrared spectroscopy was used to investigate the existence of functional groups on the treated fabric surface. Fig. 2 presents the FTIR spectra for blank cotton (B-cotton), GO–cotton (C–GO), RGO–Ag cotton (C–RGO–Ag), blank linen (B-linen), GO–linen (L–GO), and RGO–Ag linen (L–RGO–Ag) fabrics in the range of 400–4000 cm^−1^. Fig. 2a shows the FTIR spectra of the coated cotton fabrics and Fig. 2b shows the spectra of the coated linen fabrics. In the case of the uncoated cotton fabric (B), a band appeared at 3200–3500 cm^−1^, which was assigned to O–H stretching. The characteristic bands in the range of 1500–800 cm^−1^ occurred because of the presence of C–H, O–H, C–O, and C–O–C vibrations. The uncoated linen fabric was characterized by all the cellulose peaks. Overall, the FTIR spectra of the cotton and linen fabrics showed no significant differences. Moreover, the spectra of C–GO and L–GO indicated there were considerable changes with reference to the uncoated cotton and linen fabric. The spectra of the GO-coated fabrics illustrated strong peaks at 1603 and 1609 cm^−1^ for the cotton and linen fabrics, respectively. This is a sign of the presence of the C–C stretching mode of GO sheets.^29^ Also, there was an appearance of a new peak at 2160 cm^−1^ for the cotton and linen fabrics because of the O–H stretching vibration modes that were formed due to the binding of the O–H group belonging to GO with the cellulosic fabrics. These changes are strong evidence for the successful deposition of GO on the fabric surface. In addition, new weak peaks appeared at 410 and 460 cm^−1^ for C–RGO–Ag and L–RGO–Ag, respectively. This illustrates the interactions between Ag^+^ ions and oxygen functional groups of the RGO-coated fabrics.^31^

### 4.3 Surface morphology of the fabrics

**Fig. 3:**
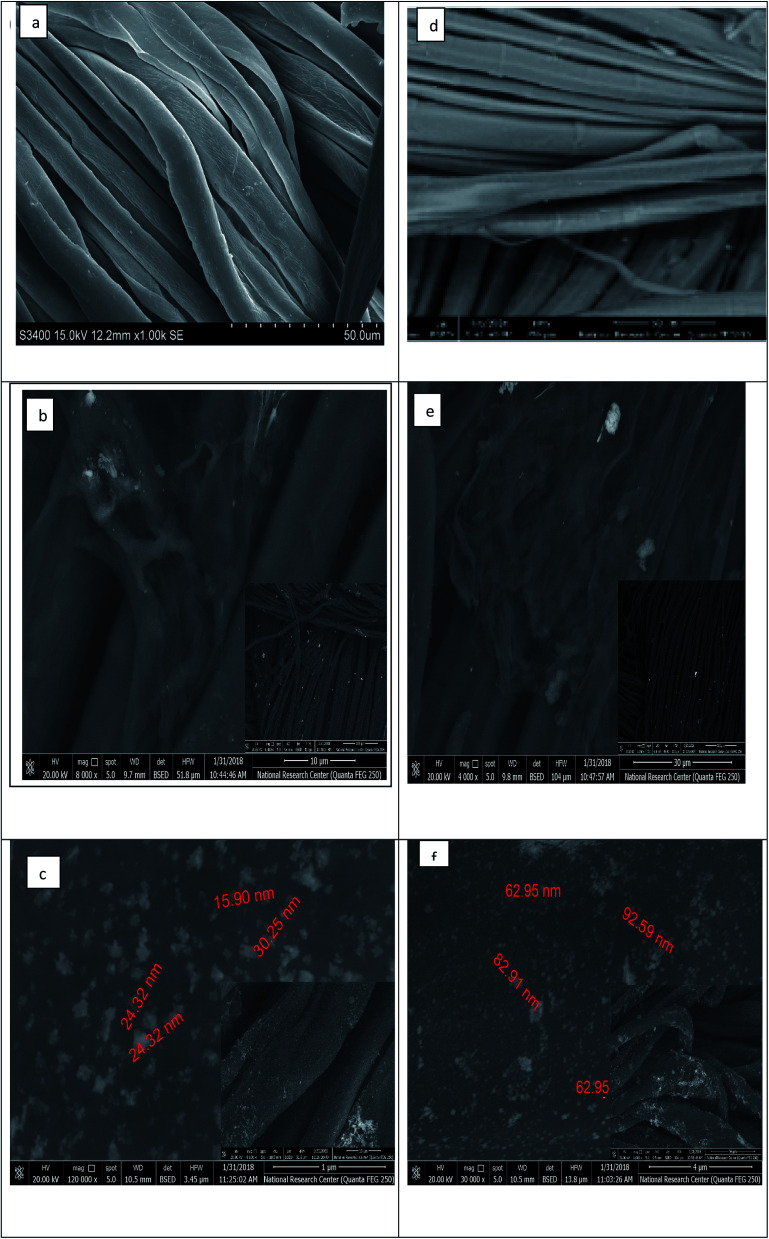
SEM images of the coated cotton and linen samples: (a, b and c) SEM images for the uncoated, GO-, and RGO/Ag-coated cotton samples; (d, e and f) SEM images for the for uncoated, GO-, and RGO/Ag-coated linen fabrics.

Characterization of the surface morphology of the uncoated and coated fabrics was performed using SEM. Fig. 3 illustrates the variation in the cotton and linen fabric's morphology. Images a, b, and c show the uncoated and coated cotton samples, while images d, e, and f are for the uncoated and coated linen fabrics. Fig. 3a and d show that the uncoated cotton and linen fabrics had fibers with a smooth surface; while Fig. 3b and e reveal the presence of GO sheets coated on the surface of the cotton and linen fabrics, respectively. The deposition of RGO/Ag nanocomposites on the surfaces of the fabrics is illustrated in Fig. 3c and f. This also indicated the presence of silver nanoparticles-decorated graphene sheets, with average sizes in the range of 15–30 nm and 26–92 nm for the cotton and linen fabrics, respectively. The *in situ* preparation of the Ag nanoparticles made them sensitive to the curing temperature. Therefore, it was not easy to keep the NPs size constant.^32^ Additionally, fabric construction may play another effective role.

**Fig. 4:**
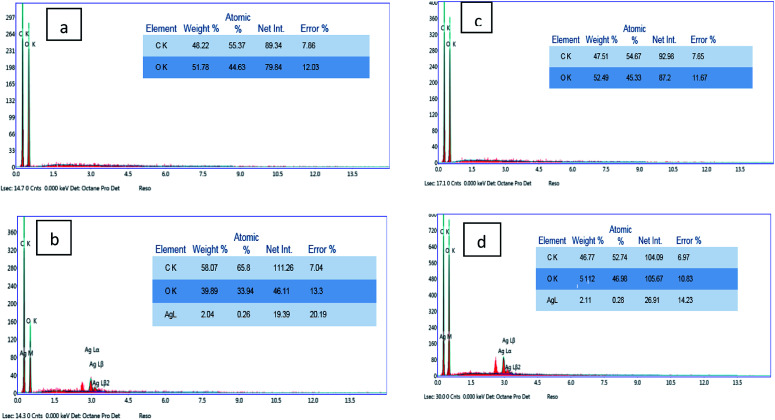
EDX analysis of the coated cotton and linen samples: (a and b) EDX for cotton coated with GO and RGO/Ag; (c and d) EDX for linen coated with GO and RGO/Ag.

The elemental composition of the coated fabrics was confirmed using EDX analysis. Fig. 4a and c show the atomic percentage of C as 48.22% along with O as 51.78% for the graphene oxide-coated cotton fabric, while the atomic percentage of C was 47.51% along with O as 52.49% for the graphene oxide-coated linen. Fig. 4b and d indicate the successful deposition of elemental Ag on the GO-coated surface of the fabrics, with weight percentages of 2.04% and 2.11% for cotton and linen, respectively. A decrease in oxygen weight percentage was clearly observed between the fabric treated with GO only (Fig. 4a and c) and the reduced graphene oxide-treated fabric (Fig. 4b and d). This outcome corresponded with several previous reports.^33^ Based on the results, the higher peaks of an observed silver element in Fig. 4d was related to a higher content of silver nanoparticles deposited on the surface of the GO-coated linen fabric compared by the lower peak of silver observed in Fig. 4b related to a lower content of silver nanoparticles deposited on the surface of the GO-coated cotton fabric.

**Fig. 5:**
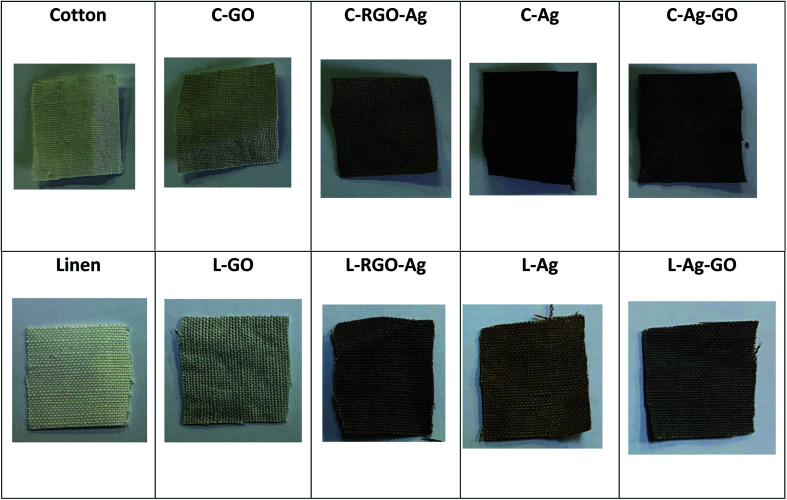
Digital photographs of the treated cotton and linen fabrics.

Color and shape in particular can be possibly utilized as a quick and instinctive path for the simple discovery and straightforward assessment of the deposition of GO and RGO/Ag nanocomposites on the cotton and linen fabric surfaces. The reflectance spectra of the digital (visual) photographs are shown in Fig. 5. The digital photographs plainly demonstrated color changes of the original fabrics after being coated with the GO sheets, Ag nanoparticles, RGO/Ag, and Ag/GO nanocomposites. The deposition of brownish-colored GO led to a change in the color of the treated fabric from white to pale brown. Additionally, the dark brown color confirmed the chemical reduction of silver ions as well as the successful deposition of RGO/Ag and Ag/GO nanocomposites on to the surfaces of the fabrics regardless of the type of substrate. The homogenous color of the RGO/Ag- compared to the Ag/GO-coated fabrics showed that reduced graphene sheets were uniformly distributed on the fabric surface and their network sheets accommodated perfectly the silver nanoparticles.

### 4.4 Antibacterial activity

The antibacterial activity of the coated fabrics with various treatments involving Ag, GO, RGO/Ag, and Ag/GO nanocomposites were assessed against representative microorganisms of open interest, both Gram-positive (*S. aureus* and *B. subtilis*) and Gram-negative (*E. coli* and *P. aeruginosa*) strains, which are extensively applied as biological indicators of pollution. The results of the antibacterial activity are summarized in Table 1.

**Table 1:** Antibacterial function and durability properties

Substrate/treatment of fabric	Inhibition zone (mm per 1 cm sample)							
	G^+^				G^−^			
	*Bacillus subtilis*		*Staphylococcus aureus*		*Escherichia coli*		*Pseudomonas aeruginosa*	
(No. of washing cycle)	1	20	1	20	1	20	1	20
C–Ag	11	9	12	9	14	12	12	10
C–GO	9	8	13	11	12	10	12	10
C–RGO/Ag	14	11	15	13	15	14	14	11
C–Ag/GO	9	8	12	9	12	10	12	10
L–Ag	15	11	16	13	16	13	16	13
L–GO	13	11	14	12	15	12	14	12
L–RGO/Ag	16	13	18	16	18	16	17	14
L–Ag/GO	10	8	12	9	13	10	12	10

The experimental results mentioned in Table 1 indicated that the linen treated fabrics showed higher antibacterial efficiency than the cotton treated fabrics. All the treated cotton and linen fabrics had more significant activity against the Gram-negative bacteria than the Gram-positive bacteria strains, this may be attributed to the structural variation of the cell wall of the bacteria. As previously reported, Gram-positive bacteria have a thicker cell wall that hinders the spreading of nanoparticles and ions into the cytoplasm.^31^ Additionally, Ag bonded easily to the cell wall of the Gram-negative bacteria more than the Gram-positive bacteria as a result of the unique outer membrane of the Gram-negative bacteria.^31^ The hindrance *versus* the pathogenic strains showed the following order:RGO–Ag > Ag > GO > Ag–GO.

The significant level of antibacterial activity of RGO/Ag nanocomposites is related to the presence of silver nanoparticles.^30^ The improvement in the activities might be recognized as being due to the modulation or adsorption of the RGO nanosheets by Ag ions, since silver nanoparticles were placed on the surface of the RGO platform, such that they were directly exposed to the bacterial cells. The GO sheets prohibited the aggregation and enlargement of silver nanoparticles; thus enhancing the composite performance. Moreover, the abrupt edges of RGO directly bound with the bacterial cell, leading to membrane tension, which brought about superoxide anion independent oxidation, and thus prompted the oxidation of nucleic acid, proteins, and lipids; thereby eventually damaging the cell membrane and causing cell destruction. Hence then, it is expected that the combination of silver nanoparticles with RGO nanosheets would have more favorable biological activities compared to the other nanomaterials acting individually. In the Ag/GO-treated samples, GO sheets enclosed the silver nanocomposite, so their release and free movement are limited, and thus they showed less antibacterial efficiency.

On the other hand, the durability toward washing was assessed according to the ASTM standard test method (D 737-109 96). The results showed that increasing washing cycles up to 20 was accompanied by a slight decrease in the antibacterial properties of the washed fabrics. This reflects the suitable applied fixation conditions.

### 4.5 UV protection properties

To study the UV-radiation-protection character of the blank fabrics and nano-/or nanocomposite-coated cellulosic fabrics, the UPF (ultraviolet protection factor) values, defined as the ultraviolet light transmittance percentage, were measured and the results are illustrated in Table 2. It was obvious that the calculated UPFs of the blank cotton and linen were 4.7 and 6.5, respectively; the difference in the structure caused this slight variation. The UPF values of the coated fabrics varied from 55.2 to 100.8 for the cotton fabrics, and from 54.3 to 98.2 for the linen fabrics, which were much higher than that of the untreated fabrics. UPF values for all the treated fabrics were far beyond the excellent protection UPF rating (50+) in the Australian/New Zealand Standard AS/NZ 4399:1996. As compared to the pure cotton and linen fabrics, a significant increase in the UPF value was observed in the case of the GO and RGO-coated fabrics. Under the influence of the good UV absorbance character of GO,^29^ coating with RGO–Ag and Ag–GO nanocomposites marginally increased the UPF values. The spaces in the coated samples prevented penetration of the UV radiation among the fabric yarns and brought about outstanding protection.^7,13^ Moreover, the large UPF values for the RGO/Ag-treated fabric reflected the super protective character of GO sheets as an outer coating layer.

**Table 2:** UPF values of cellulosic fabrics treated under different conditions

Substrate/treatment	UPF value	UV-A	UV-B	UV protection	
**Blank cotton**	7.5	22.3	18.7	5	Non-ratable
C–GO	72.2	7.4	6	50+	Excellent
C–Ag/GO	100.8	1.1	1.2	50+	Excellent
C–Ag	55.2	1.2	1.3	50+	Excellent
C–RGO/Ag	86.5	2.3	1.9	50+	Excellent
**Blank linen**	4.5	27.1	19.1	0	Non-ratable
L–GO	65.8	6.6	6.1	50+	Excellent
L–Ag/GO	98.2	1.5	1.1	50+	Excellent
L–Ag	54.3	2.6	2.3	50+	Excellent
L–RGO/Ag	83.3	2.7	2.2	50+	Excellent

### 4.6 Photocatalytic activity

**Fig. 6:**
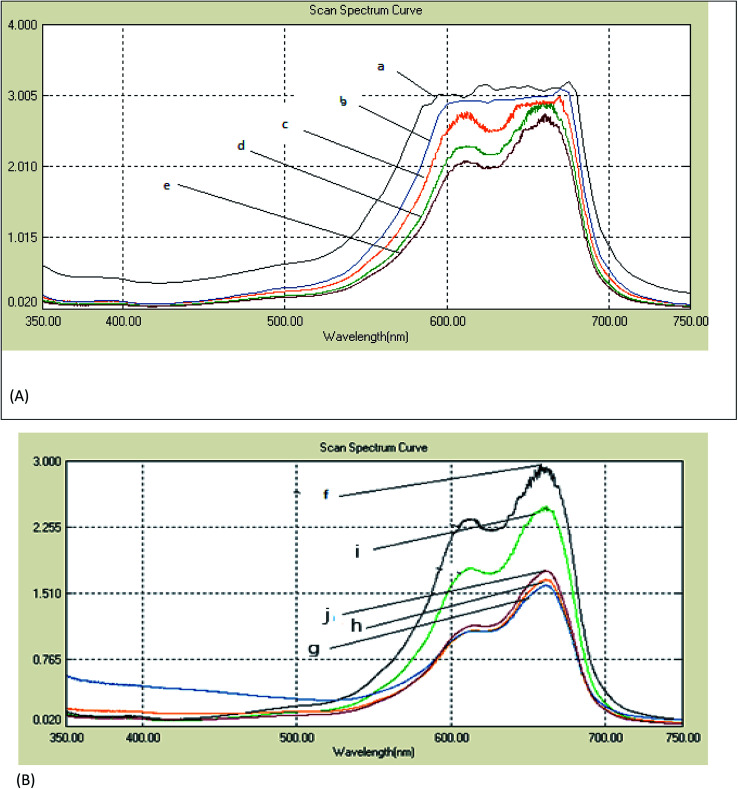
UV-vis absorption spectra from the degradation of methylene blue (MB) under a normal laboratory environment. Photodegradation of MB-treated (A) cotton fabric with the time of exposure of 12 h: (a) MB/untreated cotton fabric, (b) MB/GO-coated cotton fabric, (c) MB/Ag nanoparticle-coated cotton fabric, (d) MB/Ag–GO-coated cotton fabric, and (e) MB/RGO–Ag-coated cotton fabric. Photodegradation of MB on the treated (B) linen fabric when the time of exposure was 12 h: (f) MB/untreated linen fabric, (i) MB/GO-coated linen fabric, (j) MB/Ag nanoparticles-coated linen fabric, (h) MB/Ag–GO-coated linen fabric, and (g) MB/fabric RGO–Ag-coated linen fabric.

The spectral changes obtained by the degradation of MB adsorbed on cotton and linen fabrics are presented in Fig. 6A and B. The degradation rate for MB was recorded with respect to the change in the intensity of the absorption peak in the visible region. As shown in Fig. 6A and B, in comparison, the adsorption of MB over each of the cotton and linen fabrics (Fig. 6a and f) was insignificant. However, under visible light irradiation, in the presence of fabrics coated with the nanocomposites (C–Ag–GO, C–RGO–Ag, L–Ag–GO, L–RGO–Ag) rapid MB degradation was observed. The photocatalytic activity of the fabrics coated with Ag nanoparticles and GO sheets is also shown in Fig. 6c, j, b and i. Clearly, it can be seen that all the samples coated with the nanocomposite photocatalysts displayed a considerably improved photocatalytic effectiveness compared to those coated with Ag nanoparticles or pure graphene oxide. It was observed (Fig. 6A and B) that the rates of degradation of the adsorbed MB dye on the treated samples (regardless of the type of substrate used) followed the order:RGO–Ag nanocomposite > Ag–GO nanocomposite > Ag nanoparticles > GO sheets > MB adsorbed fabrics.

The enhancement in the photocatalytic activity of the RGO–Ag nanocomposite-treated fabrics was due to the 2D structure and large surface area of graphene oxide. Reduced graphene oxide sheets have been demonstrated to provide a better network for entrapping Ag nanoparticles, while the GO-based nanocomposites improved the adsorption capacity. Further, the high surface area of graphene sheets beside its offset face-to-face π–π interaction between the aromatic regions MB dye molecules encouraged the adsorption of a greater number of dye molecules on their surfaces.^34,35^ Here, upon exposure to visible light, the electrons below the Fermi level of the Ag nanoparticles will be excited to the surface plasmon states and the surface electrons could then be easily transferred to the conduction band of graphene oxide, leaving holes behind. RGO could act as an electron acceptor and transporter to prevent the recombination rate of the photo-generated electron–hole pairs. The electrons then convert the dissolved oxygen molecules in the reacting medium into oxygen anion radicals, and at the same time, the holes can react with adsorbed water to produce hydroxyl radicals (˙OH).^36^ The strong oxidation reactivity of O^2−^ and ˙HO2 radicals easily photodegrade MB dye. Therefore, RGO–Ag nanocomposite may act as a stable and efficient photocatalyst for the degradation of MB under sunlight irradiation.^37^ Meanwhile, the strong oxidizing potential of Ag holes could oxidize OH^−^ into OH˙ radicals that will further oxidize and decompose organic dyes.^38^ It is worthy to note that treated linen samples were more efficient in the photodegradation than the cotton samples. This could be interpreted in terms of an alternation in the chemical structure of the fabrics and hence the dye adsorption increased, leading to a higher photodecomposition of the dye.

### 4.7 Analysis of the conductivity

**Fig. 7:**
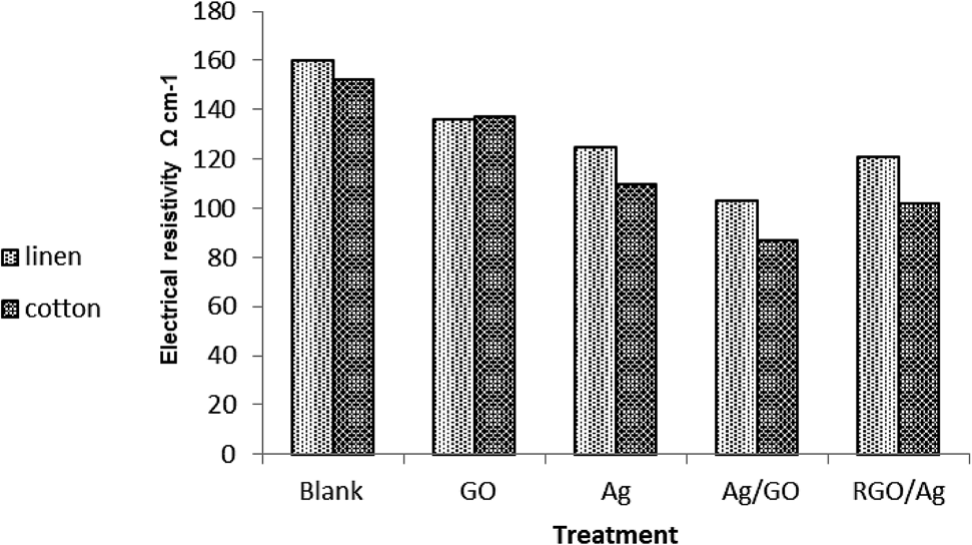
Electrical conductivity of the treated and untreated fabrics.

Fig. 7 shows the resistivity of the cotton fabric as well as of the linen fabric coated with Ag nanoparticles and GO in different sequences. It was noticed that the deposition of GO onto fabric samples led to a decrease in its electrical surface resistivity value compared with the control fabrics. This is in spite of the electrical insulation character of GO.^8^ It was found that the resistivity of both kinds of treated fabric (cotton, linen) followed the same rule. It followed the order:GO sheets > Ag nanoparticles > RGO–Ag nanocomposite > Ag–GO nanocomposite

The enhancement of the electrical conductivity of the treated fabric was ascribed to the homogenous distribution of silver nanoparticles within the GO network through permitting more active sites; thus improving the degree of electron conduction among the silver nanoparticles. Additionally, the presence of a GO sheet prevented the aggregation of silver nanoparticles, which may weaken the electrical conductivity. This proves the dual function character of GO or RGO as a stabilizer and linker or by forming a protective layer on the silver nanoparticle surface.^39^ Moreover, treatment of the fabric by Ag/GO composites caused a significant increase in the electrical conductivity compared to treatment by RGO/Ag. Besides, the electrical properties of the cotton treated fabric exceeded that of the linen treated fabric. It is noteworthy that our treated fabric was shown to be sufficient to serve as a new potential commodity in smart, next-generation wearable electronic textiles.

### 4.8 Thermal properties

**Fig. 8:**
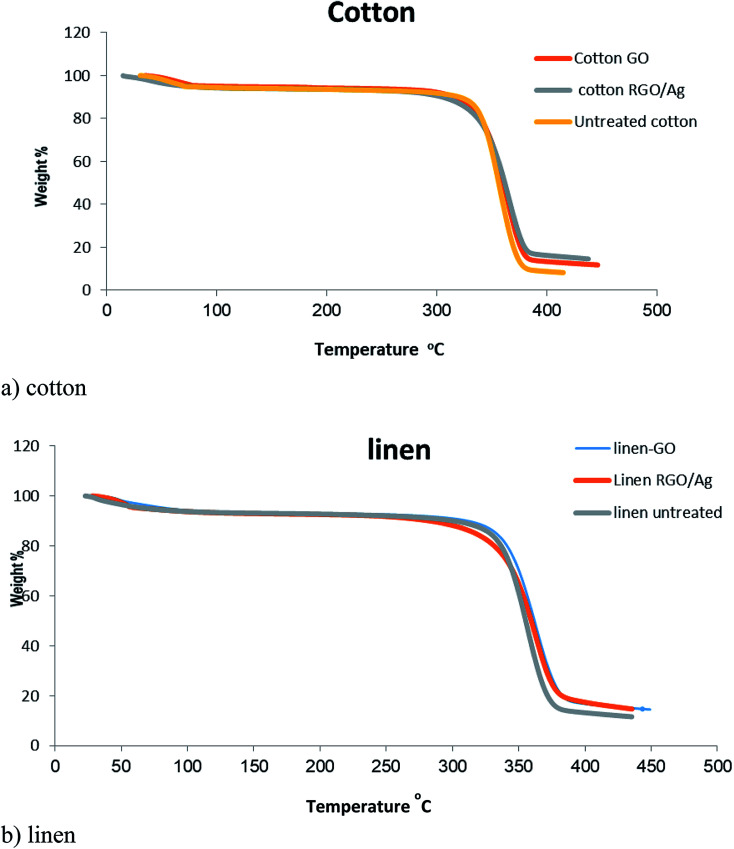
TGA curves of the untreated, GO-coated, and RGO/Ag-coated: (a) cotton fabrics, (b) linen fabrics.

Fig. 8 presents the TGA curves of blank cotton, blank linen, GO-, and RGO/Ag-treated fabrics. A similar character was observed for all the curves of the samples. There was an initial weight loss at a temperature below 100 °C due to the evaporation of water adsorbed on the fabrics. Moreover, significant weight loss in the temperature range of 300–400 °C occurred from the degradation of the cotton fabric. It's obvious from Fig. 7a that the residues of the RGO/Ag-treated cotton samples were greater than for the GO-treated samples because of the existence of silver nanoparticles adsorbed on the RGO nanosheets. This agrees with the results in previous reports. The same held true for the linen treated fabric.^40^

### 4.9 Tensile strength

Table 3 shows the effect of different treatments by graphene oxide and reduced graphene oxide on the tensile strength of the treated fabrics. The results signified that a coating of cellulosic-based fabric (cotton or linen) by GO slightly affected the tensile strength of the treated fabric, while the reduction process occurred *via* sodium tri-citrate during the synthesis of silver nanoparticles or the synthesis of graphene oxide led to a notable decrease in tensile strength.

**Table 3:** Tensile strength of cotton and linen fabrics treated with different conditions by GO, RGO, and Ag nanoparticles

Sample	Tensile strength, kg f
**Blank-cotton**	50
C–GO	52
C–Ag/GO	48
C–Ag	46
C–RGO/Ag	42
**Blank-linen**	83
L–GO	80
L–Ag/GO	75
L–Ag	76
L–RGO/Ag	73

## 5. Conclusion

In summary, we reported the successful *in situ* preparation silver nanoparticles on GO-coated fabrics to produce RGO/Ag nanocomposites as well as the coating of Ag-treated fabric with GO nanosheets to produce Ag/GO nanocomposites on the fabric. It was concluded that the sequence of treatment affected the prepared composite character. The prepared nanocomposites-coated fabric was confirmed using FTIR, SEM, and EDX. The results of the photocatalytic activity tests were shown by the reduction of methylene blue dye under UV irradiation. The RGO/Ag nanocomposite showed higher degradation rates of the adsorbed MB dye on cotton fabric compared with on linen. The results of the UV protection tests showed excellent properties when using Ag/GO nanocomposites. These results reflect the super protective character of GO sheets as an outer coating layer. Moreover, the antibacterial activity of the nanocomposite-coated fabrics presented the utmost efficiency against Gram-negative bacteria than against Gram-positive ones; while the RGO/Ag nanocomposite coating achieved a much better antibacterial character. The electrical conductivity results revealed that the synergistic effects of the Ag/GO coatings resulted in a higher electrical conductivity of the treated fabrics.

## Conflicts of interest

There are no conflicts to declare.

## Supplementary Material
